# Dosimetric benefits of deep inspiration breath-hold in postoperative radiotherapy for left-sided breast cancer after breast-conserving surgery: an anatomical parameter-based analysis

**DOI:** 10.3389/fonc.2026.1917416

**Published:** 2026-10-19

**Authors:** Yanli Cui, Shuai Xu, Xiaozhen Wang, Liwei Huo, Jintao Wen, Lixia Hao, Xiaotong Wang, Qin Zhang, Honghong Duan

**Affiliations:** Department of Radiation Oncology, Xingtai People’s Hospital, Xingtai, China

**Keywords:** cardiopulmonary dose, deep inspiration breath-hold technique, dosimetric analysis, left-sided breast cancer, radiotherapy

## Abstract

**Objective:**

To investigate the dosimetric impact of deep inspiration breath-hold (DIBH) technique on radiotherapy after breast-conserving surgery for left-sided breast cancer, and to explore the relationship between patient anatomical characteristics and dosimetric benefits of DIBH, providing preliminary evidence for understanding individual differences in DIBH related dosimetric gains and their personalized application.

**Methods:**

This retrospective study included 17 patients who underwent postoperative radiotherapy after breast-conserving surgery for left-sided breast cancer. All patients underwent computed tomography (CT) simulation under both DIBH and free-breathing (FB) conditions, and intensity-modulated radiation therapy (IMRT) plans were generated from both image sets. With identical prescription doses and planning optimization objectives, dosimetric parameters for the left lung, heart, and cardiac substructures were compared between the two respiratory conditions. Correlations of the maximum cardiac margin distance (MCMD), cardiac displacement, cardiac displacement volume, heart-to-breast distance (HBD), and changes in lung volume with dosimetric parameters were also analyzed.

**Results:**

Compared with FB, DIBH significantly reduced the mean dose (Dmean), V20, and V30 of the left lung, as well as the cardiac Dmean, V20, V30, and V40 (all *P* < 0.05). In addition, DIBH significantly reduced the mean doses to of the right ventricle (RV), left ventricle (LV), and left anterior descending coronary artery (LAD) compared with FB (all *P* < 0.05). Correlation analysis demonstrated that MCMD under the DIBH condition was positively correlated with cardiac Dmean. The change in MCMD between DIBH and FB was negatively correlated with cardiac and ventricular dose parameters. Cardiac displacement volume between the two respiratory states was positively correlated with changes in mean heart dose. Further analysis revealed that changes in lung volume were positively correlated with cardiac displacement along the Z-axis and changes in HBD while negatively correlated with LAD Dmean under the DIBH condition.

**Conclusion:**

DIBH can reduce radiation exposure to the left lung, heart, and selected cardiac substructures while satisfying target-dose requirements. Exploratory analyses indicated that cardiac geometric parameters and changes in lung volume were associated with selected dosimetric parameters, suggesting that dynamic anatomical changes during DIBH may help explain interindividual differences in dosimetric benefit. These findings require further validation in larger, multicenter cohorts.

## 1 Introduction

Breast cancer (BC) is one of the most commonly diagnosed malignancies worldwide. According to the global cancer statistics for 2022 released by the International Agency for Research on Cancer (IARC) in 2024, breast cancer accounts for approximately one-quarter of newly diagnosed cancers and one-sixth of cancer-related deaths among women worldwide (1), representing a major threat to women’s health. Previous studies have demonstrated that cardiac radiation exposure during radiotherapy for left-sided breast cancer is associated with an increased risk of subsequent ischemic heart disease. Darby et al (2) reported that the risk of ischemic heart disease increased proportionally with the mean cardiac dose, with an estimated increase of approximately 7.4% per 1 Gy increment in mean heart dose. Therefore, minimizing radiation exposure to the heart and lungs while maintaining adequate target coverage and tumor control is of considerable clinical importance.

DIBH is an established respiratory management technique designed to reduce cardiopulmonary irradiation during left-sided breast cancer radiotherapy. By increasing lung volume, lowering the diaphragm, and altering the spatial relationship between the heart, chest wall, and radiation fields, DIBH can effectively reduce the radiation dose delivered to the heart and ipsilateral lung. Previous clinical studies and meta-analyses have demonstrated that, compared with FB, DIBH significantly decreases doses to the heart, LAD, and ipsilateral lung without compromising target volume coverage. Consequently, DIBH has become an important cardiac-sparing strategy in radiotherapy for left-sided breast cancer (3–5).

However, the dosimetric benefits achieved with DIBH vary among individuals, and not all patients obtain the same degree of cardiac sparing. Recent studies have therefore focused on identifying anatomical factors that may predict the magnitude of benefit from DIBH. The research focus has gradually shifted from determining whether DIBH is effective to understanding which factors influence its effectiveness. Existing evidence suggests that patient-specific anatomical characteristics may affect the spatial relationship between the heart and irradiated regions during DIBH, thereby influencing the resulting dosimetric benefits. Parameters such as the distance between the heart and chest wall, the extent of cardiac involvement within the radiation field, and changes in cardiac position may be associated with reductions in cardiac dose after DIBH (6–8). More recently, increasing attention has been paid to dynamic changes in intrathoracic anatomy during DIBH, with evidence suggesting that inspiration-induced increases in lung volume and changes in cardiac position may be associated with the actual dosimetric benefit (5, 7).

Despite these advances, several limitations remain in current investigations of factors influencing DIBH benefits. First, previous studies have primarily focused on overall dosimetric differences between DIBH and FB, whereas the relationship between individual anatomical changes and dosimetric improvements remains insufficiently investigated. Second, some studies have relied mainly on single two-dimensional anatomical parameters, which may not fully characterize the complex three-dimensional spatial changes occurring during DIBH. Therefore, further exploration of the relationship between anatomical alterations induced by respiratory state changes and dosimetric benefits may improve understanding of the cardiac-sparing mechanisms of DIBH and facilitate its individualized application.

Therefore, this study was designed using a paired within-patient comparison between FB and DIBH conditions. In addition to evaluating the differences in dosimetric parameters of the left lung, heart, and cardiac substructures between the two respiratory states, we further investigated the associations between anatomical changes and dosimetric benefits achieved with DIBH. Specifically, anatomical parameters including MCMD, cardiac displacement, cardiac displacement volume, HBD, and lung volume changes were analyzed to explore their potential relationships with cardiopulmonary dose reductions. This study aimed to provide further insight into the anatomical mechanisms underlying the cardiac-sparing effects of DIBH and to provide a theoretical basis for individualized application of DIBH in patients with left-sided breast cancer undergoing radiotherapy.

## 2 Materials and methods

### 2.1 Patient characteristics

This retrospective study included 17 patients with left-sided breast cancer who underwent postoperative radiotherapy after breast-conserving surgery at Xingtai People’s Hospital between July 2023 and July 2024. All patients had stage I disease and met the indications for postoperative radiotherapy. The patients ranged in age from 31 to 65 years, with a median age of 49 years. This study was approved by the institutional ethics committee, and all procedures were conducted in accordance with institutional and ethical standards.

### 2.2 Inclusion and exclusion criteria

#### 2.2.1 Inclusion criteria

(1) patients with left-sided breast cancer who underwent breast-conserving surgery and required postoperative radiotherapy; (2) patients who were able to cooperate with the DIBH procedure, including a breath-hold duration of more than 35 seconds, an inspiratory volume of >1 L, and the ability to reproducibly perform the maneuver 6–12 consecutive times; and (3) patients with good treatment compliance.

#### 2.2.2 Exclusion criteria

(1) inability to cooperate with breath-holding due to cough, poor physical condition, or other reasons; and (2) pre-existing cardiac diseases, including acute or chronic heart failure, arrhythmia, coronary heart disease, acute or previous myocardial infarction, valvular heart disease, cardiomyopathy, pericardial disease, or congenital heart disease.

### 2.3 Radiotherapy equipment and treatment planning system

Radiotherapy was delivered using a medical linear accelerator (Elekta-156575, Elekta, Sweden). CT simulation was performed using a GE Discovery RT CT simulator (GE Healthcare, USA). Treatment planning was performed using the Monaco treatment planning system (Elekta, Sweden). The DIBH procedure was implemented using the Active Breathing Coordinator (ABC) system version 3.0 (Elekta, Sweden).

### 2.4 Deep inspiration breath-hold procedure using the active breathing coordinator system

The Active Breathing Coordinator (ABC) system is a respiratory motion management system used for DIBH, which enables real time monitoring of respiratory flow and control of inspiratory volume, allowing patients to maintain a reproducible deep inspiration state during CT simulation and radiotherapy. Before simulation, all patients received DIBH training. Only patients who achieved the predefined inspiratory volume and breath-hold duration requirements proceeded to formal CT simulation and treatment. According to established criteria, patients were required to maintain a breath-hold duration exceeding 35 seconds, achieve an inspiratory volume greater than 1 L, and reproducibly complete 6–12 breath-hold cycles. The ABC procedure consisted of the following steps: (1) Respiratory monitoring and signal acquisition: Patients wore a nasal clip to prevent nasal breathing and breathed through a mouthpiece connected to the ABC system. Respiratory flow changes were continuously monitored, inspiratory volume was calculated, and dynamic respiratory curves were generated in real time. (2) Individualized inspiratory threshold setting: Before treatment, an individualized inspiratory volume threshold was established based on each patient’s maximum inspiratory capacity, generally corresponding to 60–70% of the maximum inspiratory volume. After training, patients entered the breath-hold phase when the preset inspiratory threshold was reached. (3) Breath-hold maintenance and treatment delivery: After reaching the preset inspiratory threshold, the ABC system automatically occluded the breathing pathway through a valve mechanism, maintaining a stable lung volume and minimizing anatomical variations caused by respiratory motion. CT acquisition or radiation delivery was performed after confirmation of stable breath-hold conditions. (4) Safety control: when the preset breath-hold duration was reached or the patient released the handheld control button, airway occlusion was immediately released, normal breathing resumed, and radiation delivery was terminated to ensure patient safety.

### 2.5 CT simulation, target delineation, and treatment planning

Patients were positioned supine on a breast board with both arms elevated above the head. The left hand held the positioning support bar, while the right hand operated the breath-control device. The external contour of the affected breast and surgical scar were marked using radiopaque wires. CT simulation was first performed under DIBH conditions with patients positioned at the predefined inspiratory volume threshold. Subsequently, FB CT simulation was performed using the same patient positioning and scanning range. The scanning range extended from the level of the mastoid process superiorly to the inferior border of the liver inferiorly, with a slice thickness of 5mm. The two image datasets were labeled as CT-DIBH and CT-FB and imported into the Monaco treatment planning system.

The same radiation oncologist (with senior professional qualification) delineated target volumes and organs at risk (OARs) on both CT datasets to ensure consistency. The clinical target volume (CTV) included the ipsilateral breast and pectoralis major fascia. The planning target volume (PTV) was generated by expanding the CTV by 1.0 cm in the superior-inferior direction and 0.5 cm in other directions, with the skin-side margin reduced to 0.5 cm beneath the skin surface. The tumor bed boost volume was defined as follows: the gross tumor volume of the tumor bed (GTVtb) included the postoperative tumor bed; the clinical target volume boost (CTVboost) was generated by expanding GTVtb isotropically by 1.0 cm, including the surgical scar while being constrained within the CTV; and the planning target volume boost (PTVboost) was created by expanding CTVboost by 0.5 cm while remaining within the PTV. The prescribed doses were: PTV: 50 Gy in 25 fractions (2.0 Gy/fraction), with 95% of PTV receiving the prescription dose; PTVboost: 60 Gy in 25 fractions (2.4 Gy/fraction), with 95% of PTVboost receiving the prescription dose.

Two IMRT plans were generated independently by the same medical physicist based on the CT-FB and CT-DIBH datasets. Beam arrangements, weighting, and target coverage criteria were kept consistent between the two plans to ensure comparability. IMRT plans were generated using 6-MV photon beams with 6–9 fixed fields. Beam angles ranged from 290° to 160°, and the collimator angle was aligned parallel to the longitudinal axis of the target volume to reduce OAR exposure. Dose calculations were performed using the Monte Carlo algorithm in Monaco version 6.2 with a calculation grid size of 3 mm.

### 2.6 Evaluation of cardiac anatomical parameters

MCMD was measured as follows: two tangent points at the inferior border of the planning target volume (PTV) were connected, and the CT slice on which the heart was closest to the left chest wall was selected. A line parallel to the connecting line between the two tangent points was drawn through the cardiac contour, and the perpendicular distance between the two lines was measured. The measured distance was defined as MCMD. A positive value was assigned when the connecting line of the two PTV tangent points passed through the heart, whereas a negative value was assigned when the connecting line did not intersect the heart (**Figure 1B**). Cardiac displacement was evaluated based on the three-dimensional coordinate system of the treatment planning system. The X axis represented the left–right direction (leftward positive and rightward negative), the Y axis represented the superior–inferior direction (superior positive and inferior negative), and the Z axis represented the anterior–posterior direction (anterior positive and posterior negative). By comparing the coordinates of the cardiac geometric center between DIBH and FB conditions, the displacement of the heart along the three axes was calculated (**Figure 2**). HBD was measured on the coronal positioning images. The inferior border of the breast marked by the wire was used as the reference point. Combined with the three-dimensional positioning lines, the distance between the lowest point of the breast and the lowest point of the heart was measured on the coronal plane. A positive value was assigned when the inferior border of the breast was located above the inferior border of the heart, whereas a negative value was assigned when it was located below the inferior border of the heart. A value of zero was assigned when both structures were located at the same horizontal level (**Figure 1A**).

**Figure 1:**
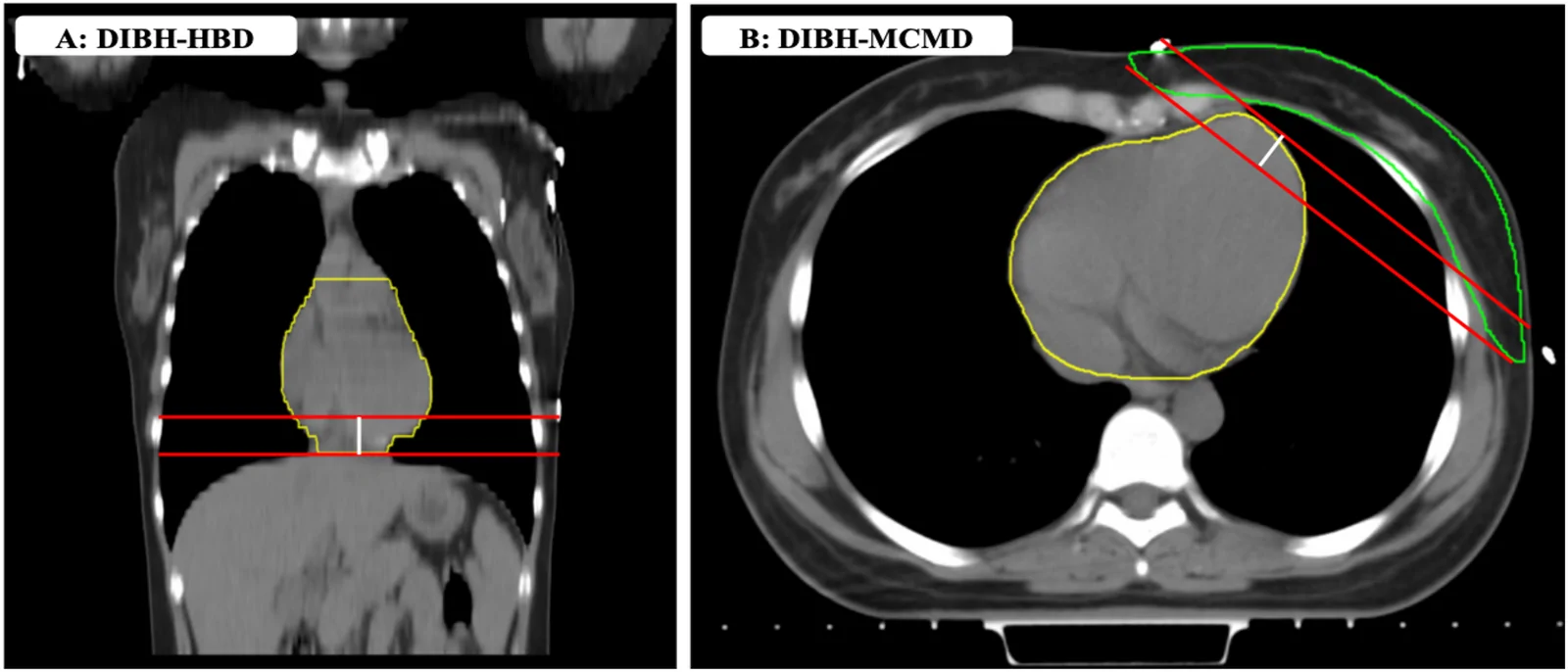
Anatomical parameters for MCMD and HBD measurement. **(A)** Measurement of heart-to-breast distance (HBD) under DIBH conditions. The inferior border of the breast and the inferior border of the heart were identified on the coronal positioning CT images using three-dimensional positioning lines. The perpendicular distance between these two reference points was measured on the coronal plane (white line). A positive value was assigned when the inferior border of the breast was located above the inferior border of the heart, a negative value was assigned when it was located below the inferior border of the heart, and zero was assigned when both structures were located at the same horizontal level. The same method was applied to measure HBD under FB conditions. **(B)** Measurement of MCMD under DIBH conditions. The CT slice where the heart was closest to the left chest wall was selected using the three-dimensional positioning line. The two tangent points at the inferior border of the PTV were connected, and a line parallel to this connecting line was drawn through the cardiac contour. The perpendicular distance between these two lines was measured as MCMD (white line). A positive value was assigned when the connecting line passed through the heart, whereas a negative value was assigned when it did not. The same method was applied for MCMD measurement under FB conditions. The yellow contour represents the heart; the red lines represent the reference and parallel lines; the white line represents the measured distance; and the green contour represents the PTV.

**Figure 2:**
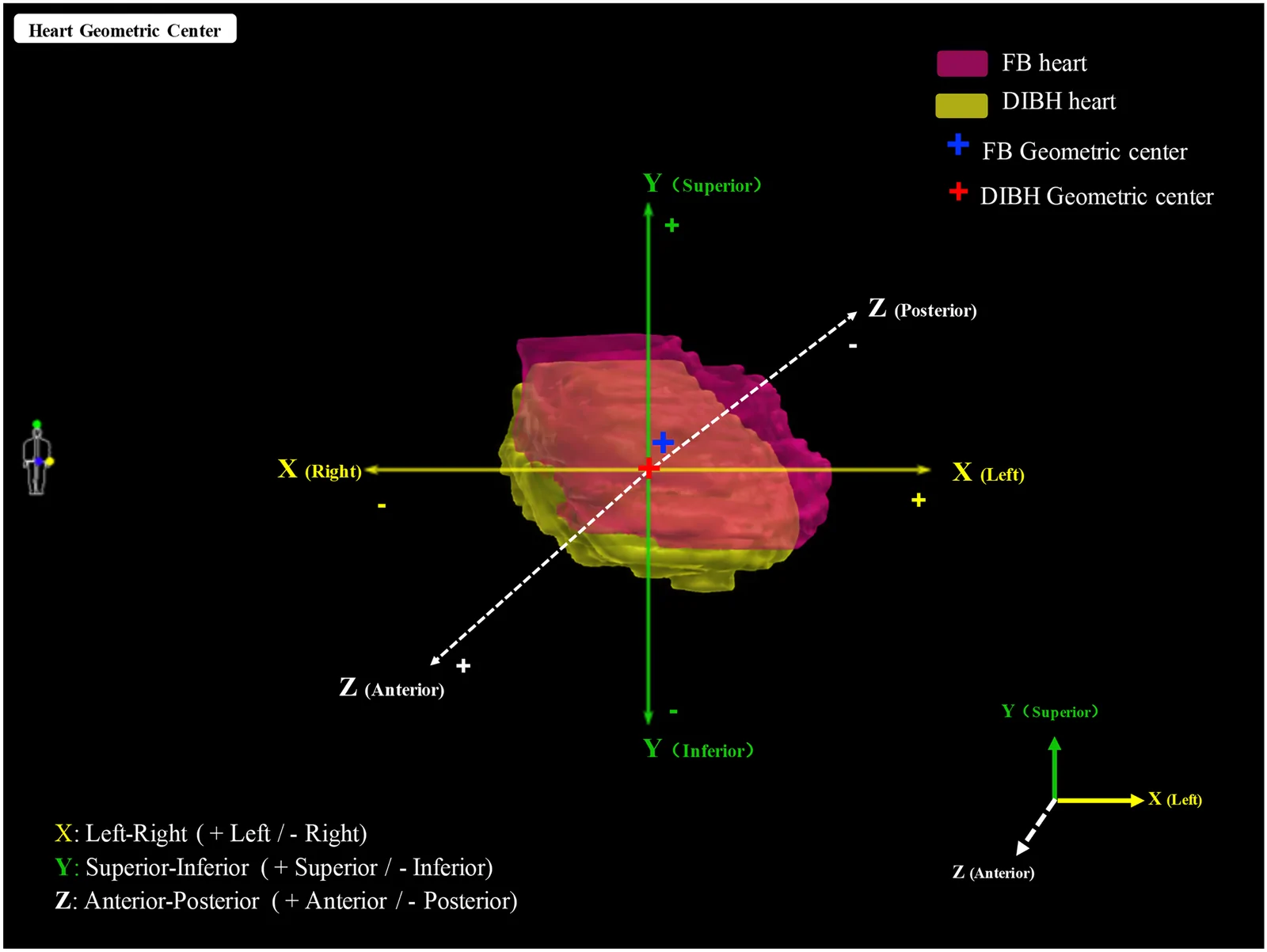
Three-dimensional displacement of the cardiac geometric center and corresponding X, Y, and Z-axis directions under DIBH and FB conditions. The figure illustrates the displacement of the cardiac geometric center between DIBH and FB conditions, including the positions of the cardiac geometric centers and the three-dimensional coordinate system used for displacement evaluation. The purple structure represents the heart under FB conditions, and the dark yellow structure represents the heart under DIBH conditions. The blue cross (+) indicates the cardiac geometric center under FB conditions, whereas the red cross (+) indicates the cardiac geometric center under DIBH conditions. The green, yellow, and white arrows represent the Y, X, and Z axis displacement directions, respectively.

### 2.7 Statistical analysis

Statistical analyses were performed using SPSS version 26.0. The normality of continuous variables was assessed using the Shapiro–Wilk test, and all variables showed a normal distribution. Data were expressed as mean ± standard deviation (mean ± SD). Paired t-tests were used to compare dosimetric parameters of the lungs, heart, and cardiac substructures between DIBH and FB conditions within the same patients. Pearson correlation analysis was performed to evaluate associations between changes in anatomical parameters and changes in dosimetric parameters. As an exploratory analysis, no correction for multiple comparisons was applied. All statistical tests were two-sided, and P < 0.05 was considered statistically significant.

## 3 Results

### 3.1 Comparison of left lung dosimetric parameters between DIBH and FB conditions

Compared with FB, DIBH significantly reduced the radiation dose to the ipsilateral lung. The Dmean of the left lung was significantly decreased under DIBH compared with FB (11.61 ± 1.02 Gy vs 12.69 ± 1.76 Gy, *P* = 0.020). Similarly, the V20 and V30 of the left lung were significantly lower with DIBH than with FB (V20: 20.42 ± 2.20% vs 22.46 ± 3.92%, *P* = 0.042; V30: 15.11 ± 2.18% vs 16.93 ± 3.18%, *P* = 0.034). Although the left lung V5 showed a decreasing trend under DIBH, the difference did not reach statistical significance (46.26 ± 4.75% vs 49.81 ± 9.23%, *P* = 0.098) (**Table 1**).

**Table 1:** Comparison of dosimetric parameters between DIBH and FB conditions.

Parameter	DIBH	FB	*P* value
	(Mean ± SD)	(Mean ± SD)	
Left lung			
Dmean (Gy)	11.61 ± 1.02	12.69 ± 1.76	0.020
V5 (%)	46.26 ± 4.75	49.81 ± 9.23	0.098
V20 (%)	20.42 ± 2.20	22.46 ± 3.92	0.042
V30 (%)	15.11 ± 2.18	16.93 ± 3.18	0.034
Heart			
Dmean (Gy)	5.02 ± 0.90	5.83 ± 1.10	0.004
V20 (%)	4.45 ± 2.28	5.83 ± 1.51	0.001
V30 (%)	2.78 ± 1.77	3.82 ± 1.30	0.001
V40 (%)	1.39 ± 1.14	1.94 ± 0.90	0.002
Cardiac substructure			
LV Dmean (Gy)	6.40 ± 1.62	7.61 ± 1.34	0.004
RV Dmean (Gy)	4.58 ± 1.12	5.53 ± 1.58	0.013
LAD Dmean (Gy)	18.65 ± 5.75	22.76 ± 4.78	0.002

DIBH, deep inspiration breath-hold; FB, free breathing; Dmean, mean dose; LAD, left anterior descending coronary artery; LV, left ventricle; RV, right ventricle; V5–V40, percentage

### 3.2 Comparison of cardiac dosimetric parameters between DIBH and FB conditions

DIBH resulted in significant reductions in radiation exposure to the whole heart compared with FB. The mean heart dose was significantly decreased under DIBH (5.02 ± 0.90 Gy) compared with FB (5.83 ± 1.10 Gy, *P* = 0.004). Furthermore, the irradiated cardiac volumes receiving moderate-to-high doses were significantly reduced: Heart V20: 4.45 ± 2.28% vs. 5.83 ± 1.51%, *P* = 0.001; Heart V30: 2.78 ± 1.77% vs. 3.82 ± 1.30%, *P* = 0.001; Heart V40: 1.39 ± 1.14% vs. 1.94 ± 0.90%, *P* = 0.002. Overall, these results demonstrate that DIBH effectively reduces both mean cardiac dose and the volume of the heart exposed to clinically relevant radiation doses (**Table 1**, **Figure 3**).

**Figure 3:**
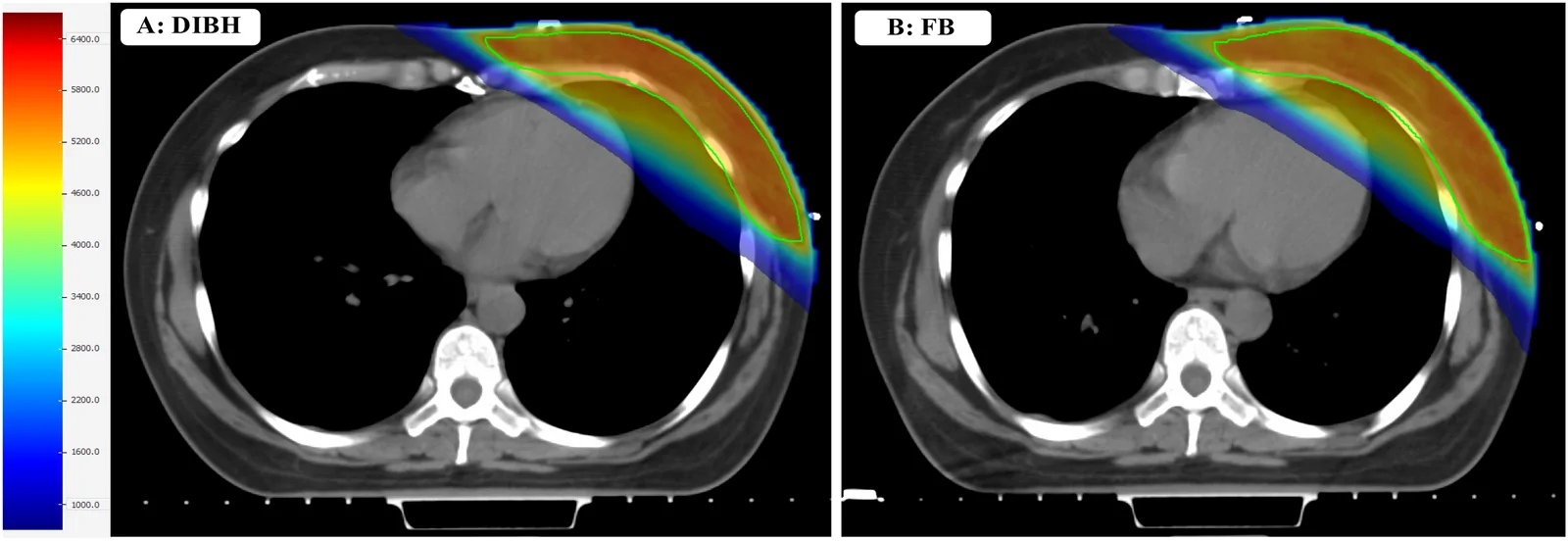
Axial CT dose distribution of IMRT-based radiotherapy for left-sided breast cancer. **(A)** Dose distribution under DIBH conditions. **(B)** Dose distribution under FB conditions. The green contour represents the planning target volume (PTV). The color scale indicates the dose distribution, with red representing high-dose regions, followed by gradually decreasing dose levels, and blue representing low-dose regions. The gray region represents soft tissue, including the heart; the black region represents lung tissue; and the white structures represent bones (vertebrae and ribs). Compared with FB, DIBH produced thoracic expansion and increased the spatial separation between the heart and the chest-wall target in this representative patient.

### 3.3 Comparison of cardiac substructure dosimetric parameters between DIBH and FB conditions

The dose distributions of major cardiac substructures were also compared between DIBH and FB conditions. DIBH significantly reduced the mean doses received by the LV, RV, and LAD. The mean doses were: LV Dmean: 6.40 ± 1.62 Gy under DIBH vs. 7.61 ± 1.34 Gy under FB (*P* = 0.004); RV Dmean: 4.58 ± 1.12 Gy under DIBH vs. 5.53 ± 1.58 Gy under FB (*P* = 0.013); LAD Dmean: 18.65 ± 5.75 Gy under DIBH vs. 22.76 ± 4.78 Gy under FB (*P* = 0.002). Therefore, DIBH provided significant dose reduction not only for the whole heart but also for specific cardiac substructures associated with radiation-induced cardiac injury (**Table 1**).

### 3.4 Correlation analysis between anatomical parameters and dosimetric changes

Correlation analyses were performed to explore the relationship between anatomical changes induced by DIBH and dosimetric benefits. Under the DIBH condition, MCMD was positively correlated with heart Dmean: (r = 0.610, *P* = 0.009). However, the change in MCMD between DIBH and FB conditions showed significant negative correlations with several cardiac dose parameters, including: Heart Dmean (r = -0.516, *P* = 0.034); Heart V20 (r = -0.538, *P* = 0.026); Heart V30 (r = -0.578, *P* = 0.015); Heart V40 (r = -0.575, *P* = 0.016). Furthermore, the change in MCMD was negatively correlated with the mean doses of both ventricles: LV Dmean (r = -0.563, *P* = 0.018); RV Dmean (r = -0.566, *P* = 0.018). These findings suggest that a greater increase in MCMD, representing greater separation between the heart and chest wall, was associated with lower cardiac exposure during DIBH.

Under DIBH, the heart shifted mainly along the Z-axis toward the chest wall, with displacement ranging from 2.5 to 27.6 mm (mean: 9.08 mm). The displacement along the X-axis ranged from -0.6 to 4.9 mm (mean: 1.60 mm), while displacement along the Y-axis ranged from -6 to 20 mm (mean: 8.58 mm). Correlation analysis demonstrated that cardiac displacement along the Z-axis was negatively correlated with heart Dmean (r = -0.504, *P* = 0.039). The cardiac displacement volume between DIBH and FB conditions ranged from 62.09 to 191.49 ml, with a mean value of 114.42 ml. Cardiac displacement volume was positively correlated with the difference in heart Dmean between DIBH and FB conditions (r = 0.689, *P* = 0.002). These results suggest that greater cardiac displacement during DIBH was associated with greater reduction in cardiac dose.

The mean difference in lung volume between DIBH and FB conditions was 1314 ± 417 mL. Correlation analysis showed that lung volume change was: Positively correlated with cardiac Z-axis displacement (r = 0.534, *P* = 0.027); Positively correlated with cardiac displacement volume (trend only, r = 0.476, *P* = 0.054); Negatively correlated with LAD Dmean under DIBH (r = -0.525, *P* = 0.031); Positively correlated with changes in heart-to-breast distance (r = 0.529, *P* = 0.029). The associations between MCMD changes, cardiac displacement, cardiac displacement volume, lung volume changes, and reductions in cardiac or cardiac substructure doses are presented in **Figure 4**. The correlation coefficients (r) and P-values are provided in each correlation plot.

**Figure 4:**
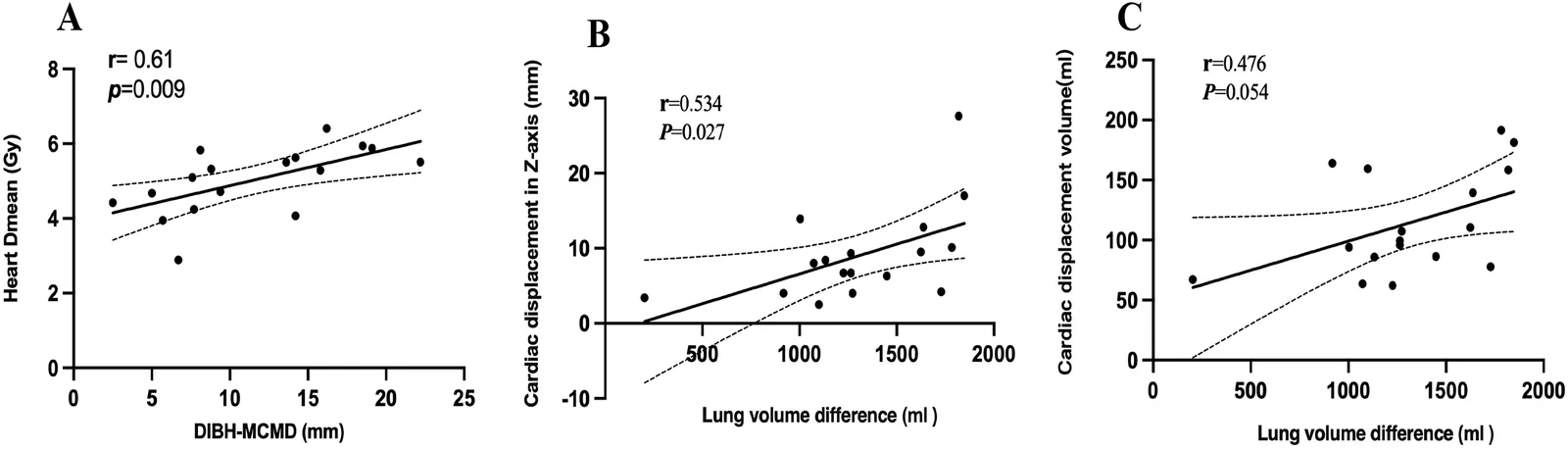
Correlations of anatomical parameters and their changes with dosimetric parameters. Each panel shows the Pearson correlation coefficient **(r)** and P-value. The solid line represents the linear regression fit. **(A)** Correlation analysis between DIBH-induced MCMD increase and heart Dmean reduction. **(B)** Correlation analysis between lung volume difference and cardiac displacement along the Z-axis during DIBH. **(C)** Correlation analysis between lung volume difference and cardiac displacement volume during DIBH.

## 4 Discussion

In this study, a paired study design was adopted, in which each patient served as their own control, to compare the dosimetric differences in cardiopulmonary structures and major cardiac substructures between FB and DIBH conditions during postoperative radiotherapy for left-sided breast cancer after breast-conserving surgery. Furthermore, we explored the potential association between anatomical changes induced by DIBH and the corresponding dosimetric benefits. Our results demonstrated that, compared with FB, DIBH significantly reduced radiation exposure to the left lung, whole heart, and major cardiac substructures, including the LV, RV, and LAD, without compromising target volume coverage. These findings further confirm the dosimetric advantages of DIBH in reducing radiation exposure to normal tissues during left-sided breast cancer radiotherapy. Furthermore, we found that changes in MCMD, cardiac displacement along the Z-axis, and lung volume expansion during DIBH were significantly associated with specific cardiac dosimetric parameters. These findings suggest that individual anatomical variations may influence the magnitude of dosimetric benefit achieved with DIBH. Consistent with previous studies, our results support the cardiopulmonary sparing effects of DIBH in patients with left-sided breast cancer. Importantly, this study extends previous findings by further exploring the potential relationship between patient-specific anatomical changes and individualized dosimetric benefits derived from DIBH. Such anatomical parameters may provide valuable information for optimizing patient selection and facilitating individualized respiratory management strategies in breast cancer radiotherapy.

Regarding lung dosimetry, our study demonstrated that DIBH significantly reduced the Dmean, V20, and V30 of the left lung, whereas the reduction in V5 did not reach statistical significance. These findings are generally consistent with previous studies. Misra et al (9) reported that DIBH could effectively reduce radiation exposure to both the lung and heart in patients with left-sided breast cancer. Similarly, Vikström et al (10) and Hjelstuen et al (11)demonstrated that DIBH decreased the dose received by the irradiated breast tissue and adjacent normal organs during tangential irradiation while maintaining adequate target volume coverage. Previous studies have suggested that pulmonary dosimetric parameters, including lung Dmean and V20, are independent risk factors associated with the development of radiation-induced lung injury (12, 13). Therefore, the reduction in left lung dose observed in our study may have potential clinical implications for reducing pulmonary toxicity after radiotherapy. However, due to the relatively small sample size of this study and the lack of evaluation of clinical pulmonary outcomes after radiotherapy, the present findings should be interpreted cautiously. Further prospective studies with larger cohorts and long-term follow-up are required to determine whether the dosimetric advantages of DIBH can translate into a reduced incidence of radiation-induced lung injury.

Regarding cardiac protection, our study demonstrated that DIBH significantly reduced heart Dmean, as well as V20, V30, and V40, compared with FB. In addition, DIBH significantly decreased the Dmean of major cardiac substructures, including LV, RV, and LAD. Furthermore, we observed a positive correlation trend between the difference in lung volume between DIBH and FB conditions and cardiac displacement volume, although this association did not reach statistical significance (*P* = 0.054). The dosimetric improvements observed in our study are consistent with previous investigations evaluating the cardiac-sparing effects of DIBH. Knöchelmann et al (14), Lai et al (15), and Testolina et al (16) reported that DIBH could significantly reduce heart dose in patients undergoing radiotherapy for left-sided breast cancer. Furthermore, Sakyanu et al (17) demonstrated that DIBH could effectively decrease radiation exposure to LAD and the heart. Previous studies have demonstrated that cardiac exposure during breast cancer radiotherapy is associated with an increased risk of long-term ischemic heart disease and cardiovascular events (2, 18). Therefore, from a dosimetric perspective, our findings further support the value of DIBH as a cardiac sparing strategy for patients with left-sided breast cancer undergoing radiotherapy. However, it should be noted that this study did not include long-term follow-up of cardiovascular events. Therefore, the observed reduction in cardiac dose cannot be directly interpreted as a reduction in the risk of clinical cardiovascular events. Further prospective studies with longer follow-up are required to determine whether the dosimetric advantages of DIBH can translate into meaningful reductions in radiation related cardiac toxicity.

This study further investigated the relationship between MCMD and the dosimetric benefits achieved with DIBH. Under DIBH conditions, MCMD was positively correlated with heart Dmean, whereas changes in MCMD between DIBH and FB were negatively correlated with changes in heart Dmean, V20, V30, V40, and Dmean of both LV and RV. These findings suggest that, within our study cohort, the alteration of the geometric relationship between the heart and the irradiation field induced by DIBH may contribute to the extent of cardiac dose reduction. The anatomical separation achieved during DIBH may therefore represent an important determinant of individual variations in cardiac dose sparing. Mohamad et al (19) further demonstrated that differences in MCMD were associated with cardiac dose reduction achieved by DIBH. Browne et al (20) also attempted to identify patients who may derive greater benefit from DIBH based on geometric characteristics. Consistent with these previous studies, our findings support the potential role of anatomical parameters in predicting DIBH-related dosimetric benefits. Furthermore, the present study extends previous analyses by evaluating the association between MCMD changes and dose reductions in specific cardiac substructures, including LV and RV.

A key issue in the clinical use of DIBH is not whether the technique is effective, but rather the interindividual variation in the magnitude of dosimetric benefit. Previous studies have focused mainly on static anatomical parameters available under FB, such as cardiac contact distance, heart-to-chest-wall distance, MCMD, and heart volume within the treatment field. Cao et al (21) found that cardiac contact distance and heart-to-chest-wall distance under FB were associated with the magnitude of cardiac dose reduction achieved with DIBH, suggesting that simple anatomical measurements may help identify patients with potentially greater benefit. Ferdinand et al. (22) further reported that changes in the heart volume within the treatment field and in maximum heart depth were associated with DIBH-related cardiac dose reductions, emphasizing the importance of anatomical changes during transitions between respiratory conditions. In contrast to these studies, the present investigation focused on dynamic anatomical changes from FB to DIBH. Changes in MCMD were associated with several cardiac dosimetric parameters, suggesting that alterations in the spatial relationship of the heart to the chest wall and irradiated region during DIBH may influence dosimetric benefit. In addition, cardiac displacement volume was correlated with the change in heart Dmean. This exploratory finding may be relevant because DIBH does more than simply increase lung volume; its potential dosimetric benefit also depends on the post-inspiratory spatial relationship of the heart to the chest wall, breast, and treatment fields.

Compared with previous research, the incremental value of this study lies in its evaluation of dynamic anatomical changes during DIBH. Earlier studies have often assessed potential dosimetric benefit using static anatomical parameters measured under FB or a single geometric indicator. In this within-patient comparison, changes in MCMD, three-dimensional cardiac displacement, cardiac displacement volume, and lung volume were evaluated simultaneously, and their relationships with dosimetric parameters of the heart and cardiac substructures were explored. The study was not intended to establish a new clinical prediction model, but rather to investigate, from the perspective of dynamic anatomical change, potential mechanisms underlying interindividual variation in the dosimetric benefit of DIBH. Compared with a single static parameter, the joint observation of multiple anatomical parameters during respiratory-state transitions may provide a more comprehensive description of DIBH-induced intrathoracic geometric changes and generate hypotheses for the subsequent development and validation of indicators for assessing DIBH benefit.

Notably, the heart underwent three-dimensional displacement during DIBH, and Z-axis displacement was negatively correlated with heart Dmean. The difference in lung volume between DIBH and FB was also positively correlated with Z-axis cardiac displacement. These findings suggest that the increase in lung volume induced by deep inspiration may be accompanied by a change in cardiac position within the thorax, thereby altering the geometric relationship of the heart to the chest wall and breast treatment region and potentially affecting cardiac radiation exposure. Compared with a single static geometric distance, three-dimensional cardiac displacement characterizes anatomical changes during DIBH in multiple directions. However, this study cannot determine whether three-dimensional displacement reflects dosimetric benefit more accurately than conventional static parameters; validation in larger cohorts using standardized three-dimensional imaging analyses is required.

The difference in lung volume was negatively correlated with LAD Dmean under DIBH and positively correlated with the difference in HBD. The relationship between the difference in lung volume and cardiac displacement volume showed only a trend toward correlation (r = 0.476, *P* = 0.054). These results suggest that DIBH-induced changes in lung volume may be accompanied by changes in the spatial relationship between the heart and breast and by alterations in cardiac position, potentially affecting radiation exposure to cardiac substructures such as the LAD. Because the correlation between the difference in lung volume and cardiac displacement volume did not meet the prespecified threshold for statistical significance (*P* = 0.054), it should be regarded only as a trend rather than a definitive statistical association. Moreover, because multiple exploratory correlation analyses were performed without adjustment for multiple comparisons, these findings should be considered hypothesis-generating and should not be interpreted as evidence of independent predictive factors.

From a clinical research perspective, the potential value of this study lies not in reconfirming that DIBH reduces cardiopulmonary doses, but in suggesting that patient-specific dynamic anatomical changes may be associated with the magnitude of dosimetric benefit. If the relationships of changes in MCMD, cardiac spatial displacement, and lung volume with dosimetric benefit are replicated in larger independent cohorts, these parameters may inform future studies of DIBH-benefit assessment and patient stratification. However, given the small sample size and the absence of model development or validation, these findings cannot currently support patient-selection criteria, clinical decision thresholds, or recommendations regarding which patients should preferentially receive DIBH.

Several limitations of this study should be acknowledged. First, the sample size was relatively small (n = 17), and the study should therefore be considered an exploratory analysis. Consequently, the stability and reproducibility of the correlation analyses may be limited, and the observed associations require validation in larger cohorts. Second, this study focused primarily on dosimetric comparisons and did not evaluate other clinically relevant aspects of DIBH implementation, including long-term reproducibility during treatment delivery, patient compliance, treatment time, resource requirements, or acute and late toxicities. Therefore, the observed dose reductions cannot be directly interpreted as improvements in clinical toxicity outcomes or survival benefits. Third, this was a single-center study without an independent validation cohort. Therefore, the anatomical parameters identified in this study have not yet undergone external validation, and their generalizability to broader patient populations remains uncertain. Finally, multiple correlation analyses were performed in this exploratory study without strict correction for multiple comparisons. Therefore, some statistically significant findings may be subject to an increased risk of type I error and should be interpreted with caution. Future studies involving larger multicenter cohorts, standardized anatomical assessment methods, and long-term clinical follow-up are warranted to further validate the clinical value of these anatomical parameters and determine their potential role in individualized DIBH selection strategies.

## 5 Conclusion

In conclusion, DIBH reduced radiation exposure to the left lung, heart, and selected cardiac substructures during postoperative radiotherapy after breast-conserving surgery for left-sided breast cancer while satisfying target-dose requirements. Exploratory analyses showed that changes in MCMD, cardiac spatial displacement, and lung volume were associated with selected dosimetric parameters, suggesting that dynamic anatomical changes during DIBH may help explain interindividual differences in dosimetric benefit.

## Data Availability

The original contributions presented in the study are included in the article/supplementary material. Further inquiries can be directed to the corresponding author.
